# Release of Prometastatic Platelet-Derived Microparticles Induced by Breast Cancer Cells: A Novel Positive Feedback Mechanism for Metastasis

**DOI:** 10.1055/s-0037-1613674

**Published:** 2017-12-15

**Authors:** Marta Zarà, Gianni Francesco Guidetti, Daniela Boselli, Chiara Villa, Ilaria Canobbio, Claudio Seppi, Caterina Visconte, Jessica Canino, Mauro Torti

**Affiliations:** 1 Department of Biology and Biotechnology, University of Pavia, Pavia, Italy; 2 FRACTAL – San Raffaele Scientific Institute, Milan, Italy

**Keywords:** cell migration, microparticles, cancer metastasis, platelet physiology

## Abstract

Circulating platelets and platelet-derived microparticles are regulators of cancer metastasis. In this study, we show that breast cancer cells induce platelet aggregation and lead to the release of platelet-derived microparticles. Although able to cause comparable aggregation, the highly aggressive MDA-MB-231 cells were more potent than the poorly aggressive MCF7 cells in inducing platelet-derived microparticles release, which was comparable to that promoted by thrombin. MDA-MB-231 cells were able to bind and internalize both MCF7- and MDA-MB-231-induced platelet-derived microparticles with comparable efficiency. By contrast, MCF7 cells did not interact with either type of platelet-derived microparticles. Upon internalization, only platelet-derived microparticles released by platelet stimulation with MDA-MB-231 cells, but not those released upon stimulation with MCF7 cells, caused activation of MDA-MB-231 cells and promoted the phosphorylation of selected signaling proteins, including p38MAPK and myosin light chain. Accordingly, MDA-MB-231-induced, but not MCF7-induced, platelet-derived microparticles dose-dependently stimulated migration and invasion of targeted MDA-MB-231 cells. These results identify a novel paracrine positive feedback mechanism initiated by aggressive breast cancer cell types to potentiate their invasive phenotype through the release of platelet-derived microparticles.

## Introduction

The evolution of the tumor and the outcome of cancer metastatic spread is regulated by circulating blood platelets. 1 2 Cancer cells stimulate platelet activation and aggregation, and, by interacting with cancer cells in the bloodstream, platelets generate a protective shield against shear stresses and immune system. 3 4 Activated platelets protect circulating cancer cells also by downregulating NK cells response through the release of TGF-β. 5 Moreover, platelets favor cancer cell adhesion to the vessel wall and subsequent extravasation. 4 6 Importantly, platelet depletion or inhibition are associated with reduced cancer spread, both in mice and in humans. 2

Platelet-derived microparticles (PMPs) are considered potential additional players in the interplay between the hemostatic system and cancer. PMPs are small lipid vesicles, with a diameter of approximately 1 μm, released in response to many physiological stimuli and represent important mediators of intercellular communication. 7 8 PMPs typically contain proteins, nucleic acids, signaling molecules, membrane receptors, and bioactive lipids, and can deliver their bioactive content to target cells promoting different biological responses. 7 8 Some studies have proposed that PMPs can interact with cancer cells to induce their activation. In particular, PMPs can regulate cancer spread by supporting cell invasion, promoting angiogenesis, and potentiating metastasis. 9 Platelets are the main source of blood-borne microparticles and the concentration of PMPs is elevated in many types of cancerous malignancies, such as skin, lung, gastric, colorectal, and breast cancers. 10 11 12 13 The mechanisms supporting the increase of circulating PMPs in cancer patients are poorly understood. In particular, it is still unknown whether cancer cells, by interacting with platelets, can directly induce the release of PMPs, and whether cancer cell–induced PMPs can operate any feedback regulation on cancer cells phenotype.

The aim of this work was to investigate the complex relationship between cancer cells and platelets focusing on the possible role of PMPs in the support of platelets to breast cancer metastasis. To this purpose, two commonly used breast cancer cell lines (MDA-MB-231 and MCF7) were selected to investigate whether these cells induce the release of microparticles from platelets. The PMPs obtained from platelets exposed to cancer cells were quantified and their ability to regulate the aggressiveness of the same cancer cells that induced their release was analyzed. The collected results demonstrate that breast cancer cells are actually able to potentiate their own invasive phenotype by inducing the release of prometastatic PMPs.

## Materials and Methods

### Platelet Isolation and Cancer Cell Maintenance

Human blood platelets were purified from fresh buffy-coat bags using a well-standardized protocol with minor modifications. 14 Briefly, buffy-coat was diluted by adding one-third volume of a 1:9 mixture of ACD (152 mM sodium citrate, 130 mM citric acid, and 112 mM glucose) and HEPES buffer (10 mM HEPES, 137 mM NaCl, 2.9 mM KCl, and 12 mM NaHCO _3_ , pH 7.4). Diluted blood was divided in 5 mL aliquots, centrifuged at 120 *g* for 15 minutes at room temperature and the supernatant was collected. Apyrase (0.2 U/mL) and PGE _1_ (1 µM) were added, and platelets were recovered by centrifugation at 750 *g* for 15 minutes, washed with 5 mL of PIPES buffer (20 mM PIPES and 136 mM NaCl, pH 6.5), and finally resuspended gently in HEPES buffer supplemented with 1 mM CaCl _2_ , 0.5 mM MgCl _2_ , and platelet poor plasma (0.05% v/v).

MDA-MB-231 and MCF7 cells were provided by Dr. Livia Visai (Department of Molecular Medicine, University of Pavia) and Dr. Maria Grazia Bottone (Department of Biology and Biotechnology, University of Pavia), respectively. Upon thawing, MCF7 and MDA-MB-231 cells were maintained in DMEM; supplemented with 10% FBS, 2 mM L-glutamine, 100 unit/mL penicillin, and 100 µg/mL streptomycin; split every 2 days; and used for the experiments within 1 month. For the platelet stimulation experiments, cancer cells were washed with PBS, and then detached by incubation for 15 minutes at 37°C with 5 mM EDTA in PBS and gentle pipetting. Cells were recovered by centrifugation and finally resuspended at the concentration of 1 × 10 ^7^ /mL in HEPES buffer containing 5.5 mM glucose and kept at 37°C until use.

### Analysis of PMPs Release Induced by Cancer Cells

Purified platelets (3 × 10 ^8^ platelets/mL) were incubated with cancer cells (5 × 10 ^4^ cells/mL) for 30 minutes at 37°C under constant stirring. As a positive control, platelet samples were stimulated with the physiological agonist thrombin (0.2 U/mL). Tumor cell–induced platelet aggregation was monitored in a Born aggregometer. In some experiments, platelets were labeled with 3 µg/mL of carboxyfluorescein succinimidyl ester (CFSE) for 10 minutes, before incubation with tumor cells.

For PMPs isolation, platelets and cancer cells were pelleted by centrifugation (750 *g* , 15 minutes). The supernatant was recovered, and either directly analyzed by flow cytometry and fluorescence microscopy or further centrifuged at 20,000 *g* for 90 minutes to collect PMPs, which were quantified for protein content by BCA assay and used for subsequent experiments. Protein content of the different preparations was used to calibrate the amount of PMPs added to cells in all the subsequent experiments.

### Analysis of PMPs Interaction with Cancer Cells

MCF7 and MDA-MB-231 cells (5 × 10 ^4^ cells/well) were grown for 24 hours on glass coverslips placed in 12-well plate and then incubated with 30 µg/mL of MCF7-induced or MDA-MB-231-induced PMPs obtained from CFSE-labeled platelets for 4 or 18 hours. Cells were subsequently washed, fixed, and examined by fluorescence microscopy. The interaction between PMPs and cancer cells was quantified as the percentage of cells associated with fluorescent PMPs, and as the average incorporated fluorescence. The internalization of cell-associated PMPs was analyzed by confocal microscopy at the Centro Grandi Strumenti (University of Pavia) using a Leica DM IRBE Inverted Microscope and the collected images were analyzed with LAS AF software.

### Cell Viability

Cell viability was assessed by using the colorimetric MTT assay. Cancer cells were seeded in 96-well plate at a density of 5 × 10 ^3^ cells/well the day before the experiment. Increasing amounts of PMPs were incubated with cells for 24 hours at 37°C. MTT solution was added at the final concentration of 0.5 mg/mL and plate was incubated at 37° for 3 additional hours. Finally, dimethyl sulfoxide was added and, after 10 minutes at room temperature, plate was read using a test wavelength of 570 nm and a reference wavelength of 650 nm.

### Migration and Invasion Assay

The effect of PMPs on migration and invasiveness of cancer cells was evaluated using Falcon cell culture inserts (8-µm pore size) positioned in a 24-well plate. For the invasion assays, the upper side of the insert was coated with 0.1 mL of Matrigel (50 µg/mL) following the manufacturer's indications. Cancer cells were serum starved for 6 hours and then resuspended DMEM added of 2 mM L-glutamine, 100 unit/mL penicillin, and 100 µg/mL streptomycin and 0.5% FBS. Cell samples were either left untreated or treated with increasing amount of the different PMP preparations and then transferred inside the inserts. Complete medium, containing 10% FBS, was added to lower chamber. Upon 18 hours, cells that moved through the porous membrane were stained with 0.5% crystal violet and counted at 20× microscope magnification.

### SDS-PAGE and Immunoblotting

The analysis of protein expression and phosphorylation was performed by SDS-PAGE followed by immunoblotting as described. 15 The following antibodies were used: anti-phospho-Akt (Ser473), anti-phospho-Erk (Tyr202/204), anti-phospho-MLC (Ser19), anti-phospho-p38MAPK (Thr180/Tyr182; Cell Signaling), and anti-GAPDH (Santa Cruz). Quantification of band intensity was performed by computer-assisted densitometric scanning using ImageJ Version 1.42 software.

### Statistics

All the reported figures are representative of at least three experiments and data were analyzed by *t* -test (for comparisons of two groups) or one-way ANOVA with the Bonferroni post-test (for multiple comparisons).

## Results

### Cancer Cell–Induced Release of PMPs

**Fig. 1:**
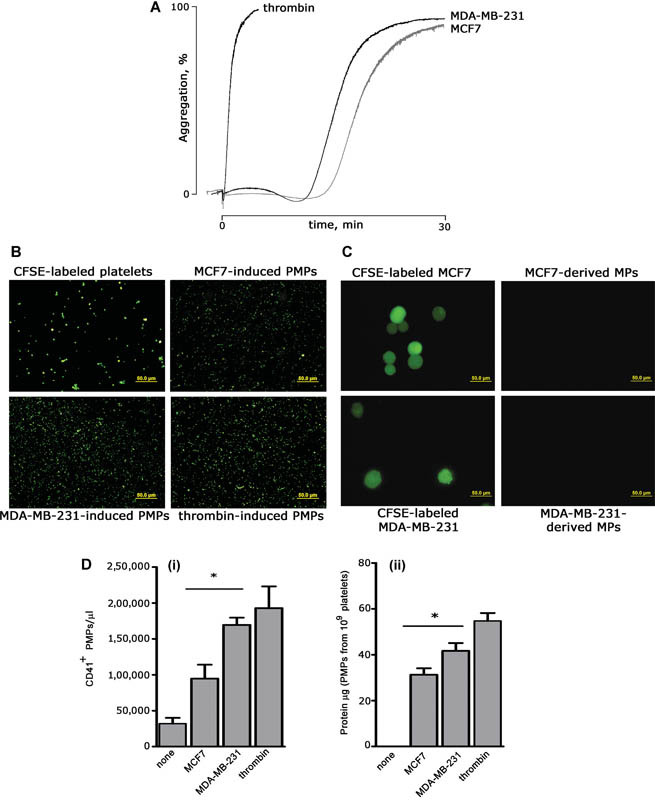
Breast cancer cells induce the release of platelet-derived microparticles (PMPs). ( **A** ) Washed human platelets (0.3 × 10 ^9^ platelets/mL) were incubated for 30 minutes at 37°C under constant magnetic stirring with 5 × 10 ^4^ /mL of either MCF7 or MDA-MB-231 cells. Thrombin was used at 0.2 U/mL. Representative aggregation traces are reported. ( **B** ) PMPs release in the supernatant. Platelets were labeled with 3 µg/mL of carboxyfluorescein succinimidyl ester (CFSE) and co-cultured with either MCF7 or MDA-MB-231 cells or stimulated with thrombin for 30 minutes at 37°C under stirring. Upon centrifugation, to remove platelets and cells, labeled PMPs in the supernatant were visualized by fluorescence microscopy. Representative images of labeled platelets and released PMPs are reported. Scale bars: 50 µm. ( **C** ) Platelets do not cause the release of breast cancer cell–derived microparticles. Experiments were performed as those in panel B, except that MCF7 and MDA-MB-231 cells were labeled with CFSE before being co-cultured with unlabeled platelets. Scale bars: 50 µm. ( **D** ) Quantification of PMPs released by platelet stimulation with MCF7, MDA-MB-231, or thrombin. (i) PMPs were quantified using a three laser-equipped BC Navios flow cytometer (Beckman Coulter), upon staining with PE-labeled anti-CD41 antibody (Bio Legend). The instrument was set using Megamix-Plus (BioCytex, Marseille, France) fluorescent calibrated beads (0.1–0.9 μm range). Data were analyzed with the FCS Express 6.0 software (De Novo Software), and are reported as mean ± SEM ( *n* = 3). * *p* < 0.05. (ii) PMPs in the supernatants were recovered by centrifugation at 20,000 *g* for 90 minutes, resuspended in HEPES buffer, and analyzed by BCA protein assay. Results report the protein content of PMPs released from the same number of stimulated platelets (10 ^9^ ) and are mean ± SEM ( *n* = 7). * *p* < 0.05.

Elevated amounts of circulating PMPs are a common feature in breast cancer patients, often associated with an advanced metastatic stage. 13 To investigate the mechanism of cancer-associated increase of PMPs, we investigated whether, in vitro, breast cancer cells themselves directly stimulate the release of microparticles from human platelets. To this purpose, we used two well-characterized metastatic breast cancer cell lines from pleural effusion: the more aggressive, triple-negative, mesenchymal-like MDA-MB-231 cells, and the less aggressive estrogen and progesterone receptors positive, epithelial-like MCF7 cells. Both MDA-MB-231 and MCF7 cells were able to activate platelets and caused a comparable irreversible platelet aggregation within 30 minutes ( Fig. 1A ). Differently from thrombin, a prolonged lag time was observed, as documented also for other cancer cell lines. 16 17 The ability of breast cancer cells to stimulate the release of PMPs was investigated by exposing platelets labeled with the fluorescent probe CFSE to MDA-MB-231 or MCF7 cells for 30 minutes. Untreated and thrombin-stimulated platelets were used as negative and positive controls, respectively. Upon removal of platelets and cancer cells by centrifugation, the supernatants were spotted on glass coverslips and analyzed by fluorescence microscopy. Fig. 1B shows that, as seen with thrombin, both MDA-MB-231 and MCF7 cells caused an evident release of fluorescently labeled microparticles from platelets. Although platelets are small cells, with an average diameter of 2 to 4 µm, the size of labeled microparticles recovered from the supernatant was clearly smaller, and was consistent with that expected for PMPs (up to 1 µm), thus confirming that no contaminant cells remained upon centrifugation. In reverse experiments, unlabeled platelets were incubated with CFSE-labeled breast cancer cells, but no fluorescent microparticles were detected in the supernatant ( Fig. 1C ), demonstrating that, when co-cultured, breast cancer cells induce the release of microparticles from platelets, but not vice versa. Accordingly, immunoblotting analysis revealed that released microparticles were CD41 positive (data not shown). Although we decided to focus the present study only on two selected breast cancer cell lines, we observed PMPs release also upon exposure of platelets to other cancer cells, such as the colorectal cancer cell line Caco2 cells and the murine melanoma cell line B16F10 (data not shown). This evidence indicates that the ability to cause the release of PMPs is likely a common feature of many cancer cells.

Quantification of breast cancer cell–induced PMPs release was performed by flow cytometry using fluorescent counting beads (Flow-Count Fluorospheres BC) and by protein content determination. Fig. 1D shows that the two cell lines were differently potent in inducing PMPs release. In fact, the more aggressive MDA-MB-231 cells were significantly the more efficient MCF7 cells in inducing PMPs release both in terms of particles number (169,533 ± 11,763 vs. 95,000 ± 22,584 PMPs/µL) and protein content (41.6 ± 3.4 µg vs. 31.2 ± 2.8 µg of protein contained in PMPs released from 10 ^9^ platelets). Interestingly, the number of PMPs released by stimulation with MDA-MB-231 cells was comparable to that induced by the physiological agonist thrombin (190,000 ± 30,292 PMPs/µL), one of the most potent inductor of microparticles release ( Fig. 1D ).

### Cancer Cell–Induced PMPs Differently Interact with Cancer Cells

Since microparticles released upon activation of platelets with physiological agonists such as thrombin are able to interact with cancer cells, 18 19 we investigated whether MCF7-induced and MDA-MB-231-induced PMPs were also capable to subsequently interact with the same cell types that promoted their release.

**Fig. 2:**
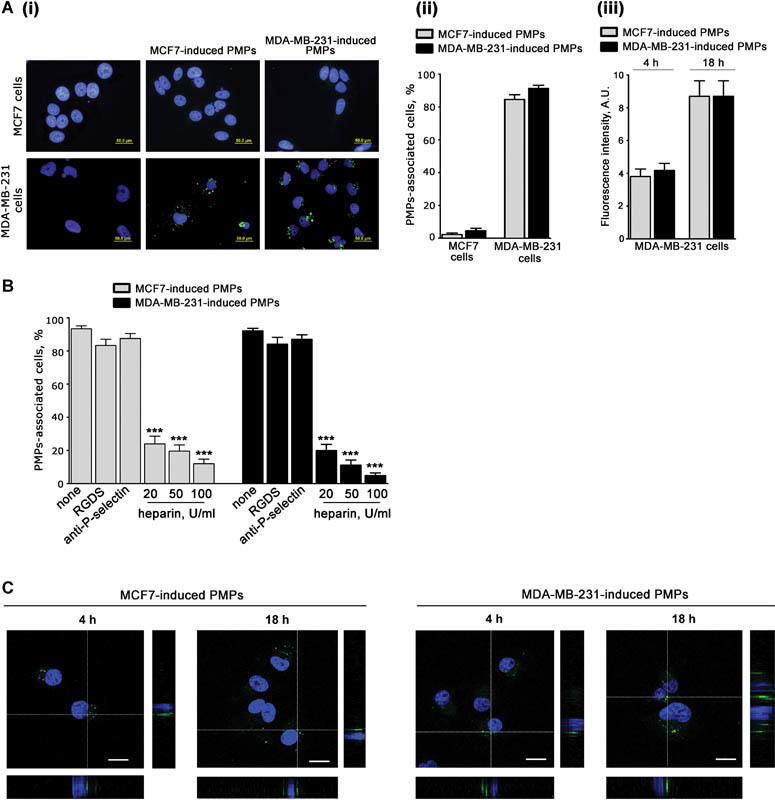
Interaction of breast cancer cells with platelet-derived microparticles (PMPs). ( **A** ) MCF7 and MDA-MB-231 cells (5 × 10 ^4^ cells/well) were grown on glass coverslips and then incubated with 30 µg/mL of MCF7-induced or MDA-MB-231-induced PMPs obtained from carboxyfluorescein succinimidyl ester (CFSE)-labeled platelets (green) for 4 or 18 hours. (i) Representative fluorescence microscopy images upon 18 hours of incubation. Cell nuclei were stained with 1 µg/mL of Hoechst (blue); (ii) quantification of the percentage of MCF7 or MDA-MB-231 cells (as indicated on the bottom) associated with fluorescent PMPs, induced by either MCF7- or MDA-MB-231 cells (gray and black bars, respectively, as indicated on the top). Data are the mean ± SEM of three independent experiments. (iii) Comparison of MDA-MB-231 cells' ability to interact with MCF7- or MDA-MB-231-induced PMPs after 4 or 18 hours of incubation. Data are expressed as average green fluorescence intensity associated with each cell and are the mean ± SEM of three independent experiments. ( **B** ) MDA-MB-231 cells were incubated with MDA-MB-231- or MCF7-induced PMPs obtained from CFSE-labeled platelets (30 μg/mL) in the presence of buffer (none), 0.5 mM RGDS, 10 μg/mL anti-P-selectin antibody CLB/thromb/6, or increasing amount of heparin, as indicated. The percentage of PMPs-associated cells was determined as described in panel A, and results are expressed the mean ± SEM of three independent experiments (*** *p* < 0.001). ( **C** ) Confocal microscopy analysis of MDA-MB-231 cells interacting with MCF7- or MDA-MB-321-induced PMPs for the indicated times of incubation. Representative confocal middle z-sections and orthogonal views are reported. Scale bars: 20 µm.

Equal amounts of fluorescent PMPs, produced by CFSE-labeled platelets treated with either MCF7 or MDA-MB-231 cells (indicated as MCF7-induced and MDA-MB-231-induced, respectively), were incubated for 18 hours with both types of breast cancer cells. More than 90% of MDA-MB-231 cells were found to be surrounded by both MCF7-induced and MDA-MB-231-induced PMPs. By contrast, less than 3% of MCF7 cells were associated with either type of PMPs ( Fig. 2A ). To compare the ability of MDA-MB-231 cells to associate with the two types of PMPs, we measured the cell-associated fluorescence. We found that MDA-MB-231 cells were equally able to interact with MCF7-induced or MDA-MB-231-induced PMPs, both at a short or at a late time ( Fig. 2A ). These results indicate that although MCF7 cells can efficiently aggregate whole platelets to support PMPs release, they are unable to subsequently interact with released PMPs. By contrast MDA-MB-231 cells efficiently interact with PMPs originated by platelets stimulated with both cell lines.

Previous studies have indicated a role for integrin αIIbβ3 and P-selectin in platelet interaction with cancer cells. 20 21 To get further insights into the mechanism of PMPs interaction with MDA-MB-231 cells, we analyzed the effect of the integrin αIIbβ3 antagonist peptide RGDS and of the P-selectin blocking antibody CLB/thromb/6 (NOVUS Biologicals). Fig. 2B shows that neither integrin αIIbβ3 nor P-selectin inhibition reduced the interaction of MDA-MB-231 cells with either type of PMPs. By contrast, we found that this interaction was dose dependently inhibited by heparin, suggesting the implication of a still unknown carbohydrate-recognizing mechanism ( Fig. 2B ).

Confocal microscopy analysis revealed that MDA-MB-231-associated fluorescent PMPs were localized on the same focal plane as cell nuclei, indicating that both MCF7- and MDA-MB-231-induced PMPs were internalized. This process occurred rapidly, being already detectable after 4 hours of incubation ( Fig. 2C ). These results indicate that the two different breast cancer cell lines, both of which are able to activate platelets to release PMPs, are differently targeted by the circulating PMPs that they have induced.

### PMPs Modulate Migration and Invasiveness of MDA-MB-231 Cells

**Fig. 3:**
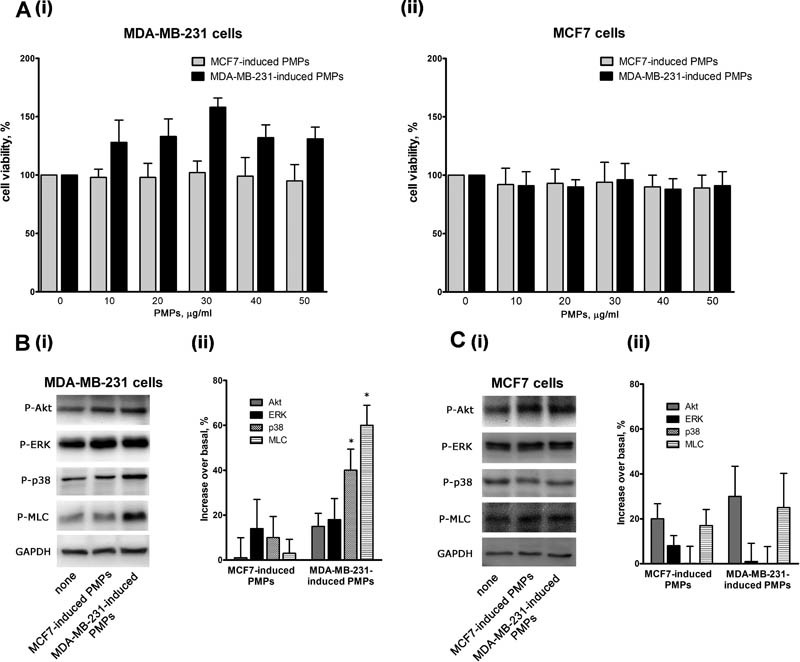
Platelet-derived microparticles (PMPs)-induced activation of MDA-MB-231 cells. ( **A** ) Viability of MDA-MB-231 (i) or MCF7 (ii) cells incubated with the indicated amounts of PMPs for 24 hours was assessed by a colorimetric MTT assay. Results are reported as the mean ± SEM of three different experiments. ( **B and C** ) Phosphorylation of selected signaling proteins in MDA-MB-231(panel B) or MCF7 (panel C) cells incubated with MCF7- or MDA-MB-231-induced PMPs for 18 hours, as indicated on the bottom. Representative immunoblot with specific anti-phosphoprotein antibodies directed against the protein indicated on the right is reported in (i), where GAPDH staining is for equal loading control. Quantification of the results by densitometric scanning is reported in (ii), as % of phosphorylation increase over the level of untreated cells. Results are the mean ± SEM of three different experiments. * *p* < 0.05.

We next investigated the functional consequence of PMPs interaction and internalization by MDA-MB-231 cells. Neither type of PMPs displayed cytotoxic effects on either MDA-MB-231 or MCF7 cells, as no alteration of cell viability was observed upon incubation with increasing amounts of PMPs (0–50 µg/mL; Fig. 3A ). The slight increase observed when MDA-MB-231 cells were incubated with MDA-MB-231-induced PMPs was not statistically significant. To investigate whether interaction with PMPs caused activation of MDA-MB-231 cells, we analyzed the phosphorylation of selected signaling proteins. Cells were incubated with identical amount of PMPs, as determined by protein content assessment, at a final PMPs concentration of 30 µg/mL. We found that only MDA-MB-231-induced, but not MCF7-induced, PMPs significantly stimulated the phosphorylation of p38MAPK and myosin light chain (MLC) in MDA-MB-231 cells ( Fig. 3B ). This effect was selective, as phosphorylation of other intracellular signaling molecules, such as Akt or ERK, was unaffected. Consistent with the previously shown inability to bind and internalize PMPs, protein phosphorylation in MCF7 cells was unaffected by the addition of both types of PMPs ( Fig. 3C ).

**Fig. 4:**
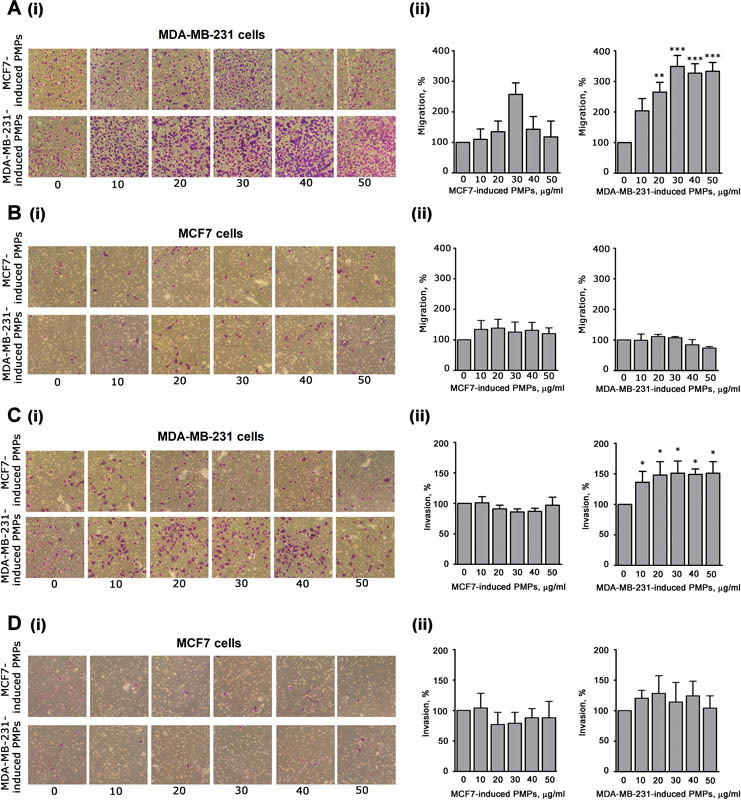
Analysis of cell migration and invasion. Effect of MCF7- or MDA-MB-231-induced PMPs (as indicated on the left) on migration (panels A and B) and invasiveness (panels C and D) of MDA-MB-231 cells (panels A and C) or MCF7 cells (panels B and D), as indicated. Cancer cells were treated with increasing amounts of the two different types of PMPs preparations (0–50 μg/mL, as indicated on the bottom) and then transferred inside cell culture inserts. For the invasion assays (panels C and D), the upper side of the insert was coated with 0.1 mL of Matrigel (50 µg/mL). Incubation was prolonged for 18 hours and the cells that moved through the porous membrane were stained and counted. In all the panels, representative images are reported in (i), while quantification of the results is shown in (ii) as mean ± SEM of three experiments. Statistical significance of the difference was calculated between treated and untreated cells (sample 0). * *p* < 0.05; ** *p* < 0.01; *** *p* < 0.001.

It is known that p38MAPK and MLC are implicated in the control of cell motility. 22 We found that PMPs induced by MDA-MB-231 cells, but not those induced by MCF7 cells, strongly and dose-dependently potentiated the migration of MDA-MB-231 cells ( Fig. 4A ). This effect was particularly evident at doses of PMPs higher than 20 µg/mL, which are in the range of the microparticles concentration found in the plasma of cancer patients. 23 Both preparations of PMPs were essentially ineffective on the migration of MCF7 cells ( Fig. 4B ).

Finally, we analyzed the effect of PMPs on the ability of cancer cells to invade extracellular matrix, through a Matrigel-coated transwell assay. Fig. 4C shows that MDA-MB-231-induced PMPs promoted a significant potentiation of the invasive ability of MDA-MB-231. Again, MCF7-induced PMPs displayed no effects. No stimulation of invasion propensity of MCF7 was observed when MCF7 cells were incubated with both types of PMPs ( Fig. 4D ). These results indicate that MDA-MB-231 cells are differently targeted by PMPs released upon platelet aggregation by MDA-MB-231 or MCF7 cells.

## Discussion

In this article, we demonstrated for the first time that breast cancer cells can efficiently induce the release of microparticles from blood platelets and that the released microparticles can signal back to cancer cells to alter their invasive properties.

The ability of cancer cells to bind and activate platelets has been widely investigated. 1 24 Previous “in vitro” studies have documented the peculiar and delayed kinetics of cancer cell–induced platelet aggregation compared with physiological agonists, but still the mechanism, the signal transduction pathways, and the receptors involved in this process remain poorly characterized. Nevertheless, platelet aggregation induced by cancer cells is considered an important step for the metastatic process, as it can facilitate tumor cell survival in the circulation and favor extravasation. 4 Similarly, the release of microparticles from platelets has emerged as a novel strategy for intercellular communication, as PMPs are known to carry several biological signals that can be efficiently transferred to target cells. 7 8 Our study moves a step forward in understanding the complex interplay between cancer cells and blood platelets, as it identifies a previously unrecognized mechanism by which cancer cells exploit blood platelets to generate PMPs able to increase their metastatic potential.

The use of two different breast cancer cell lines characterized by a different metastatic phenotype also provided multiple evidences that this novel interplay between cancer cells and blood platelets is rather complex and is strongly dependent on the characteristic of the cancer cells. Both MDA-MB-231 and MCF7 cells were able to cause platelet aggregation and the release of PMPs. By contrast, cancer cell–induced platelet aggregation is not accompanied by the release of microparticles from cancer cells. Nevertheless, different cancer cells, albeit able to induce comparable platelet aggregation, are differently potent in stimulating PMPs release. This is also very well outlined in this study, as we document that MDA-MB-231 cells are more potent than MCF7 cells in inducing PMPs release, although both kind of cells cause comparable platelet aggregation. Therefore, tumor cell–induced platelet aggregation is necessary, but clearly not sufficient to induce PMPs release. The precise understanding of the mechanism for cancer cell–induced release of PMPs is largely hampered by the current poor characterization of the mechanism supporting cancer cell–induced platelet aggregation. How these events are linked and what is the role played in both processes by cell–cell contact events, rather than release of soluble factors with paracrine activity, deserve further detailed investigations. Nevertheless, the different efficiency of MDA-MB-231 and MCF7 cells to induce PMPs release is suggestive that, when released from solid tumor into the bloodstream, differently aggressive cancer cells may produce variable platelet responses.

An additional important finding of our study is that PMP released upon cancer cells interaction with platelets acts as regulators of cancer cells themselves. Few previous studies have indicated that PMPs released by platelet activation by classical agonists such as thrombin, which occurs during a hemostatic or thrombotic event, are able to activate cancer cells. 9 18 19 25 Here we show that cancer cells are active players in a novel positive feedback mechanism, as they are not only targets of PMPs but also promoters of their release independently of platelet implication in hemostasis or thrombosis.

Interestingly, only MDA-MB-231 but not MCF7 cells were able to interact with, and rapidly internalize, PMPs. This indicates that the ability to bind PMPs is not a general feature of tumor cells, but depends on the cell type. The fact that MCF7 cells induce platelet aggregation and the release of PMPs, but are unable to subsequently bind them, also indicates that the ability of cancer cells to bind PMPs is clearly distinct from the ability to bind intact platelets, and suggests significant differences in receptor recognition. However, how MDA-MB-231 cells bind to PMPs is still unclear. Our evidence indicates that this interaction does not involve either integrin αIIbβ3 or P-selectin. However, the sensitivity to heparin suggests the implication of a still unknown carbohydrate-based recognition mechanism. Further studies are certainly required for a precise characterization of the receptors involved.

PMPs released upon platelet stimulation with MDA-MB-231 or MCF7 cells are internalized by MDA-MB-231 cells with a similar efficiency. However, only PMPs generated by platelet interaction with MDA-MB-231, but not with MCF7 cells, stimulate the phosphorylation of selected signaling proteins and increase migration and invasion of MDA-MB-231 cells. Therefore, although different types of cancer cells can induce PMPs release, only some subsets of released PMPs display prometastatic effects on the generating cancer cells. These findings suggest that PMPs released by platelets in response to different types of cancer cells are intrinsically different from their ability to alter the migratory phenotype of the target cells. This is consistent with previous studies indicating that, when platelets are stimulated with physiological agonists, the composition of released PMPs may vary depending on the generating stimulus. 7 It is important to outline that, in our functional assays, identical amounts of isolated PMPs recovered by centrifugation from the platelet releasate have been used. Although the possibility that some large macromolecular complexes coprecipitate with the recovered PMPs cannot be completely rule out, the differential effects on cell migration and invasion cannot be ascribed to the presence of unidentified soluble agonists released by stimulated platelets. It is also very important to note that the stimulation of MDA-MB-231 cells migration was observed in response to a range of PMPs concentration comparable to that measured in the plasma of cancer patients, 21 indicating that the effect may be physiologically relevant.

In conclusion, our results identify a novel positive feedback mechanism by which some cancer cells may increase their own aggressiveness while circulating in the bloodstream, by inducing the release of specific PMPs. It is conceivable that, by interacting with platelets and inducing their aggregation, cancer cells generate a prometastatic niche in which high concentrations of PMPs may signal back to the generating cancer cell to alter its phenotype. The nature of the circulating cancer cell dictates the relevance of this prometastatic feedback. The identification of the molecular determinants responsible for cancer cell–PMPs interaction may be useful to develop novel pharmacological strategy to limit the efficacy of such prometastatic loop.

Although further studies are certainly required to map the ability of cancer cells from different tumors to stimulate PMPs release and to become their biological targets, our results document a novel and unexpected mechanism supporting platelet-assisted cancer metastasis.
